# Evolution of H6N6 viruses in China between 2014 and 2019 involves multiple reassortment events

**DOI:** 10.1080/22221751.2024.2341142

**Published:** 2024-04-06

**Authors:** Yingying Du, Jun Xia, Zhengxiang Wang, Jie Xu, Yanhong Ji, Yinghong Jin, Ling Pu, Shuai Xu

**Affiliations:** a State Key Laboratory for Animal Disease Control and Prevention, College of Veterinary Medicine, Lanzhou University, Lanzhou Veterinary Research Institute, Chinese Academy of Agricultural Sciences, Lanzhou, People’s Republic of China; b Institute of Veterinary Medicine, Xinjiang Academy of Animal Sciences, Urumqi, People’s Republic of China; c Guizhou Institute of Animal Husbandry and Veterinary Science, Guizhou, People’s Republic of China

**Keywords:** Influenza A virus, H6N6 subtype, reassortment, intermediate, evolution

## Abstract

H6N6 avian influenza viruses (AIVs) have been widely detected in wild birds, poultry, and even mammals. Recently, H6N6 viruses were reported to be involved in the generation of H5 and H7 subtype viruses. To investigate the emergence, evolutionary pattern, and potential for an epidemic of H6N6 viruses, the complete genomes of 198 H6N6 viruses were analyzed, including 168 H6N6 viruses deposited in the NCBI and GISAID databases from inception to January 2019 and 30 isolates collected from China between November 2014 and January 2019. Using phylogenetic analysis, the 198 strains of H6N6 viruses were identified as 98 genotypes. Molecular clock analysis indicated that the evolution of H6N6 viruses in China was constant and not interrupted by selective pressure. Notably, the laboratory isolates reassorted with six subtype viruses: H6N2, H5N6, H7N9, H5N2, H4N2, and H6N8, resulting in nine novel H6N6 reassortment events. These results suggested that H6N6 viruses can act as an intermediary in the evolution of H5N6, H6N6, and H7N9 viruses. Animal experiments demonstrated that the 10 representative H6N6 viruses showed low pathogenicity in chickens and were capable of infecting mice without prior adaptation. Our findings suggest that H6N6 viruses play an important role in the evolution of AIVs, and it is necessary to continuously monitor and evaluate the potential epidemic of the H6N6 subtype viruses.

## Introduction

Influenza A viruses (IAVs) belong to the *Orthomyxoviridae* family and contain eight segments of negative-sense single-stranded RNA [1]. Segments 4 and 6 encode the surface glycoproteins hemagglutinin (HA) and neuraminidase (NA), respectively. Segments 1, 2, and 3 encode the polymerase proteins PB2, PB1, and PA, respectively. Segment 5 encodes the nucleoprotein (NP), and segment 7 encodes the matrix protein M1 and ion-channel protein M2. Segment 8 encodes NS1 and NEP proteins [2]. To date, researchers have identified 18 HA and 11 NA subtypes of IAVs from various hosts [3]. A segmented genome facilitates gene exchange between different influenza viruses when they infect the same cell, leading to the emergence of novel reassortment viruses [4]. Reassortment viruses have been identified as the cause of four human influenza pandemics and several poultry outbreaks [5-8]. However, studies on the characteristics leading to the reassortment of viruses remain insufficient.

In 2013, China reported the first cases of H5N6 viruses with novel reassortment [9]. Since then, the World Health Organization (WHO) has reported 90 laboratoryconfirmed cases of human infection with the influenza A (H5N6) virus including 35 deaths [10]. Subsequently, it was reported that H5N6 viruses were generated from reassortment between H5N1 and H6N6 viruses, with H6N6 acting as the donor strain that provided NAs and/or internal genes to the H5N6 viruses [9]. Furthermore, Jin et al. demonstrated that the internal genes of two influenza A (H7N9) strains originate from H6N6 viruses [11]. He et al. revealed that human H7N9 viruses contribute internal genes to H6N6 viruses [12]. These results indicate that H6N6 viruses are implicated in the evolution of H5N6 and H7N9 viruses. However, more studies are still needed to better understand the evolutionary dynamics of H6N6 viruses.

Previous studies have demonstrated that H6 subtype viruses, with different NA genes from N1 to N9 in the Eurasian and North American lineages, have been isolated with increasing frequency from wild birds and domestic avian species [13,14]. A surveillance study from Northern Europe identified H6 as the most frequently detected influenza virus subtype from 1998 to 2006, which is known for its broader host range compared with other subtypes [15]. Among the three unique clades of H6 subtype viruses (ST339-like clade, ST2853-like clade, and HN573-like clade) [16], the ST2853-like clade of the H6 subtype has gradually emerged as the dominant strain lineage, exhibiting distinct antigenicity compared with the others. Since 2009, H6N6 viruses have continuously replaced H6N2 viruses as the most prevalent strains among the H6 subtypes in Eastern China [17]. Several studies have also observed that certain H6N6 viruses can infect and cause illnesses in mice, guinea pigs, and swine, posing a potential threat to human health [14,18-22].

Although sequence analysis of H6N6 viruses has been conducted, the evolutionary patterns remain unclear. In this study, we analyzed the complete genomes of 30 H6N6 viruses collected between November 2014 and January 2019 from domestic farms and live poultry markets (LPMs) in China. Phylogenetic analyses of the H6Ny HA gene showed that laboratory viruses belonged to the ST2853-like clade of the H6 Eurasian lineage, with 6 out of 30 showing a deletion of 11 amino acids in the NA genes. Through phylogenetic analysis of 198 full-length genome sequences of H6N6 strains, we identified 98 genotypes among H6N6 strains in China. Notably, the 30 isolates were found to result from reassortment events involving viruses of subtypes H6N2, H5N6, H7N9, H5N2, H4N2, and H6N8. Base compositional data from the maximum clade credibility (MCC) trees of the H6 HA and N6 NA genes indicated that the evolution of H6N6 viruses in China was not interrupted by selective pressure. Therefore, it is necessary to investigate the potential significance of low pathogenic AIVs in the ongoing emergence of highly pathogenic AIVs and novel reassortment viruses.

## Materials and methods

### Facility statement

All experiments conducted in this study adhered to the protocols approved by the Lanzhou Veterinary Research Institute (LVRI) of the Chinese Academy of Agricultural Sciences in Gansu, China. The protocols for animal studies were approved by the Committee on Ethics of Animal Experiments of LVRI [23].

### Samples and viruses

Samples used in this study were collected from November 2014 to January 2019 from ducks, geese, and the environment at domestic farms or LPMs in China’s Anhui and Hunan provinces. Cloacal and tracheal swabs from each bird were immediately placed in separate collection tubes with 1 mL of sterile viral transport medium supplemented with 8000 U/mL of penicillin and streptomycin. Samples were then inoculated into the allantoic cavities of 10-day-old, specific-pathogen-free (SPF) embryonated chicken eggs at 37°C for 48 h. The viruses underwent biological cloning through three rounds of limiting dilution in SPF chicken eggs, and virus stocks were maintained at –70°C until use [24,25]. Allantoic fluid was harvested and subjected to hemagglutination assays using 1% chicken red blood cells. Using hemagglutination inhibition (HI) assay, the subtypes of the hemagglutination-positive allantoic fluids were identified and the interference of NDV was excluded. Reverse transcription PCR (RT–PCR) was employed to further identify the subtype of influenza viruses and exclude NDV.

### Reverse transcription

Viral RNA was extracted from the hemagglutination-positive allantoic fluid using an RNA Extraction Mini Kit (TIANGEN Biotech Co. Ltd., China) and transcribed into cDNA using a universal 12-bp primer (5′-AGC AAA AGC AGG-3′) [24]. The eight viral gene segments were amplified using specific primers complementary to the conserved promoter and noncoding regions of each gene segment (primer sequences were listed in Table 1). The amplified fragments were purified using agarose gel DNA extraction (E.Z.N.A. DNA kit, Omega Bio-Tek, China) and confirmed using gel electrophoresis. DNA sequencing was performed by Tsingke Biotech Co. Ltd., China.

**Table 1.** Primers used in this study.

Target	Forward (5′–3′)	Reverse (5′–3′)
NDV NP	GATGAGCTTTGCACCTGCTG	TGCTCATAAAGTCCCTGGCG
AIV M	GACCRATCCTGTCACCTCTGAC	CCACAATATCAAGTGCAA
HA	ACAAAAGCAGGGGAAAATGA	AGTAGAAACAAGGGTGTTTTTYTCTA
NA	AGCAAAAGCAGGGTGAAAATG	TTCCWAAATCWTTTCTACTTAAAG
PB2	AGCRAAAGCAGGTCAAWTATATTCA	ACTGCYTTTATCATGCAMTCYTC
	GCAACRGCTATYYTRAGGAAAGC	AGTAGAAACAAGGTCGTTTTTAAA
PB1	AGCRAAAGCAGGCAAACCATTTGAA	GAGGATTGGAGYCCRTCCCACCA
	ATGATGATGGGCATGTTCAACATG	AGTAGAAACAAGGCATTTTTTCA
PA	AGCRAAAGCAGGTACTGATYCAAAA	ATCCARCTYGARTCWGTCAATTC
	GGCACCRGARAAAGTRGACTTTGA	AGTAGAAACAAGGTACTTTTTTGGA
NP	AGCRAAAGCAGGGTAGATAATCA	AGTAGAAACAAGGGTATTTTTCTT
M	AGCRAAAGCAGGTAGATRTTKAAA	AGTAGAAACAAGGTAGTTTTTTA
NS	AGCRAAAGCAGGGKGACAAARACA	AGTAGAAACAAGGGTGTTTTTTATCAT

### Sequence assembly

Raw reads were mapped to the influenza virus database to select the best-matched reference sequences. Subsequently, raw reads and reference sequences were corrected for mismatches based on single fluorescent label nucleotides using SeqMan software. Finally, the strain sequences were edited using the Editseq module of the DNASTAR package (version 7.1) to identify an open reading frame and translate the DNA.

### Sequence alignment

A total of 1848 HA sequences of H6Ny viruses and 3081 NA sequences of HxN6 viruses, deposited in full-length only, were obtained from the Influenza Virus Resource at the National Center for Biotechnology Information (https://www.ncbi.nlm.nih.gov, NCBI) and only complete sequences from the Global Initiative of Sharing All Influenza Data database (https://www.gisaid.org/, GISAID) until January 2019. Among the 1848 HA and 3081 NA sequences, we screened 432 H6N6 strains originating from China, each with full-length HA and NA gene segments. In addition, a subset of 168 H6N6 strains isolated from China with complete genomic sequences was aligned. The nucleotide sequences of the coding regions of each gene segment were aligned using MAFFT (version 7.304b) [26]. Sequences were corrected using Mega (version 7) or Notepad++ (version 7.5.8).

### Phylogenetic analyses

A total of 1848 HA sequences of H6Ny viruses were clustered using a 98% similarity cutoff, resulting in 54 representative sequences. Likewise, 3081 NA sequences of HxN6 viruses were clustered with a 99% similarity cutoff, leading to the identification of 57 representative sequences [27,28]. The nucleotide substitution model used the Bayesian information criterion through Model Finder in IQ-TREE (version 2.1.1). The system phylogeny trees for the large datasets were inferred from nucleotide alignment using PhyML (version 3.0) or BEAST (v1.10.4) under the nucleotide substitution model [29,30].

### Dynamic evolution

Dynamic evolutionary analysis was performed using 462 H6N6 strains originating from China, including 30 strains isolated in this study and 432 strain collected from the database. Temporal phylogenies and rates of evolution were inferred using BEAST software (v1.10.4) with an uncorrelated relaxed clock model; this allowed for rate variation among lineages within a Bayesian Markov Chain Monte Carlo (MCMC) framework [31]. Bayesian MCMC sampling analyses used chain lengths of at least 100 million steps, with a 10% “burn-in” removed. To ensure adequate sampling, multiple independent runs of each sequence were performed, compared, and combined. The resulting tree file was visualized using FigTree (version 1.4.4).

### Animal experiments

Based on reassortment events and genotype variations, 10 representative H6N6 viruses were selected. The intravenous pathogenicity index (IVPI) test in chickens with representative viruses was assessed following the WHO protocol [10]. Ten 6-week-old SPF chickens per group were inoculated with 0.2 mL of 1:10 diluted virus intravenously, testing the level of pathogenicity of an isolate by observing clinical signs in infected birds over 10 days.

Groups of 11 6-week-old female BALB/c mice were anesthetized with CO_2_ and intranasally inoculated with 10^6^ EID_50_ (50% egg infectious dose) viruses in a 50 µL volume. Three mice were sacrificed on days 3 and 5 post-infection, and the lung tissues were collected for virus titration in eggs. The weight changes and deaths of the remaining five mice in each group were monitored daily for 14 days [32].

### Statistical analysis

The base compositional data were graphically plotted using GraphPad Prism (version 3.4.0) and modified in Adobe Illustrator.

### Data  availability

The data generated or analyzed during this study are included in this paper. The sequences used in this study have been deposited in GISAID, and the accession numbers have been listed in Supplementary Data 1.

## Results

### Phylogenetic analysis of H6 and N6 AIVs

**Figure 1.**
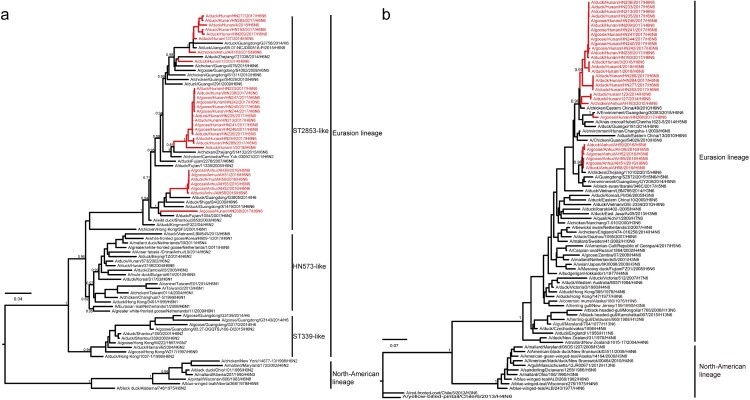
Maximum likelihood phylogenetic analysis of H6 and N6 genes. A total of 1848 H6 HA and 3081 N6 NA sequences were collected from the NCBI and GISAID databases worldwide until January 2019. Additionally, 30 isolates were collected from the Anhui and Hunan provinces of China between November 2014 and January 2019. The tree is rooted at the midpoint. The red taxon line represents the H6N6 subtype isolated in the laboratory. (a) Phylogenetic tree of the HA gene of H6 viruses. (b) Phylogenetic tree of the NA gene of N6 viruses.

Over 60,000 cloacal and tracheal swabs were collected from ducks, geese, and the local environment. From this collection, we isolated 30 H6N6 viruses along with 10 other subtypes of viruses, including H3N2, H3N6, H3N8, H6N2, H6N1, H10N5, H9N2, H5N1, H5N6, and H7N9. To explore the evolutionary relationship of the H6N6 viruses, we aligned the sequences of 1848 H6 HA genes and 3081 N6 NA genes obtained from the NCBI and GISAID databases until January 2019, along with HA and NA genes of the 30 viruses isolated from November 2014 to January 2019. We utilized CD-HIT to select representative strains and construct maximum likelihood (ML) trees. According to the phylogenetic analyses, the 30 isolates were typed as ST2853-like clade within the H6 Eurasian lineage (Figure 1a). Next, the phylogenetic tree of N6 NA genes was separated into the North-American and Eurasian lineages. All NA genes of the 30 laboratory strains were clustered into the N6 Eurasian lineage, with six out of seven Anhui strains exhibiting deletion of 11 amino acids (58–68) in the NA genes, which were absent in the Hunan strains (Figure 1b and Figure S1).

### Host distribution of the H6 subtype AIVs

**Figure 2.**
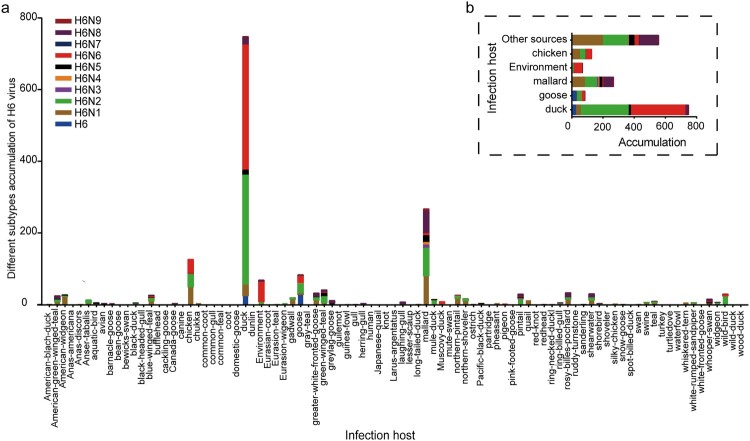
Host distribution of H6 viruses. (a) Host information for 1848 H6Ny viruses. The color key represents the subtype of H6 viruses. (b) Accumulation of H6 subtype strains isolated from different hosts.

Furthermore, the H6 subtype showed a broad host distribution, infecting 89 different host species (Figure 2a). The isolation rates of the H6 subtype AIVs varied, with percentages of 6.82% (126/1848), 40.48% (748/1848), 4.49% (83/1848), 3.68% (68/1848), 14.45% (267/1848), and 30.09% (556/1848) from chickens, ducks, geese, environmental samples, mallards, and other sources, respectively (Figure 2b). Statistically, the H6 subtype viruses were mainly isolated from waterfowls, such as ducks, geese, and mallards, which are the dominant reservoirs of H6 viruses.

### Genotype distribution of H6N6 viruses

To determine the genotype distribution of H6N6 viruses in China, all eight gene segments of 198 strains were subjected to phylogenetic analysis, which include 168 strains from databases with complete genomic sequences and 30 strains isolated in this study. By employing a bootstrap value threshold of ≥ 60 of the phylogenic trees [33], the PB2, PB1, PA, HA, NP, NA, M, and NS gene segments were clustered into 8, 7, 10, 5, 11, 11, 8, and 7 groups, respectively (Figures S2a-h and Supplementary Data 2).

**Figure 3.**
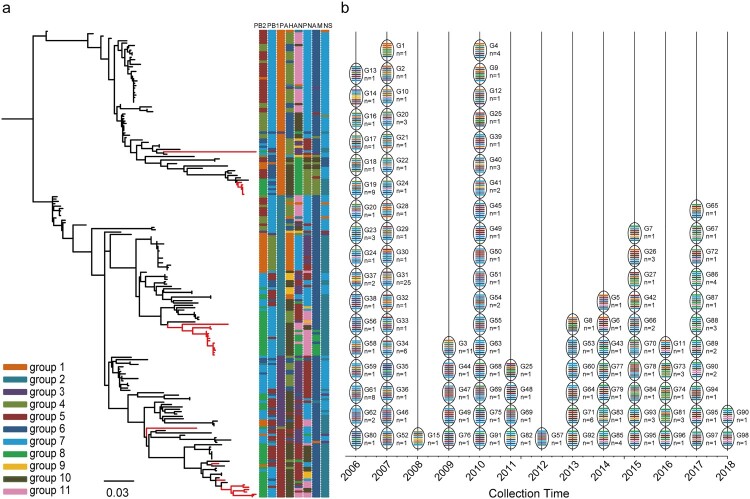
Genotypes distribution of 198 complete genomes of H6N6 viruses in China. The genotype distribution of the complete genomes of H6N6 viruses was based on a group of bootstrap values. Each vertical bar represents a gene group. (a) ML tree of H6N6 HA gene. The strains isolated in this study are highlighted in red on the phylogenetic tree. The color key represents the whole genome combined with eight gene segments. (b) Collection time distribution of H6N6 virus genotype.

Genotypes were further defined based on the group of the eight gene segments (Figure 3a and Figure S3) (referred to as “Genotype,” or G). Moreover, we designated all 198 viruses as 98 genotypes. Significantly, all the genotypes appeared in only one year or the adjacent two years (Figure 3b). For example, in 2006, G19 (n = 9) and G61 (n = 8) were typical, but G19 and G61 only distributed in 2006. There were four strains of G4, G85, and G86, but they were only detected in 2010, 2014, and 2017. The emergence and fading of genotypes indicate substantial and frequent reassortments of H6N6 viruses in China.

Supplementary Data 2 indicated that ducks are the most important reservoir for H6N6 viruses, with the largest number of genotypes (75.51% [74/98]). Among other sources, chickens accounted for 15.31% (15/98), geese for 8.16% (8/98), and environmental samples for 6.12% (6/98) of genotypes. Based on the geographical distribution of the H6N6 viruses, Fujian had 19 genotypes (62/198) and Guangdong province had 33 genotypes (49/198). Collectively, Fujian and Guangdong provinces accounted for 53.06% of the genotypes, suggesting that the two provinces played an important role in the evolution of H6N6 viruses.

### Dynamic time evolution of H6N6 viruses

**Figure 4.**
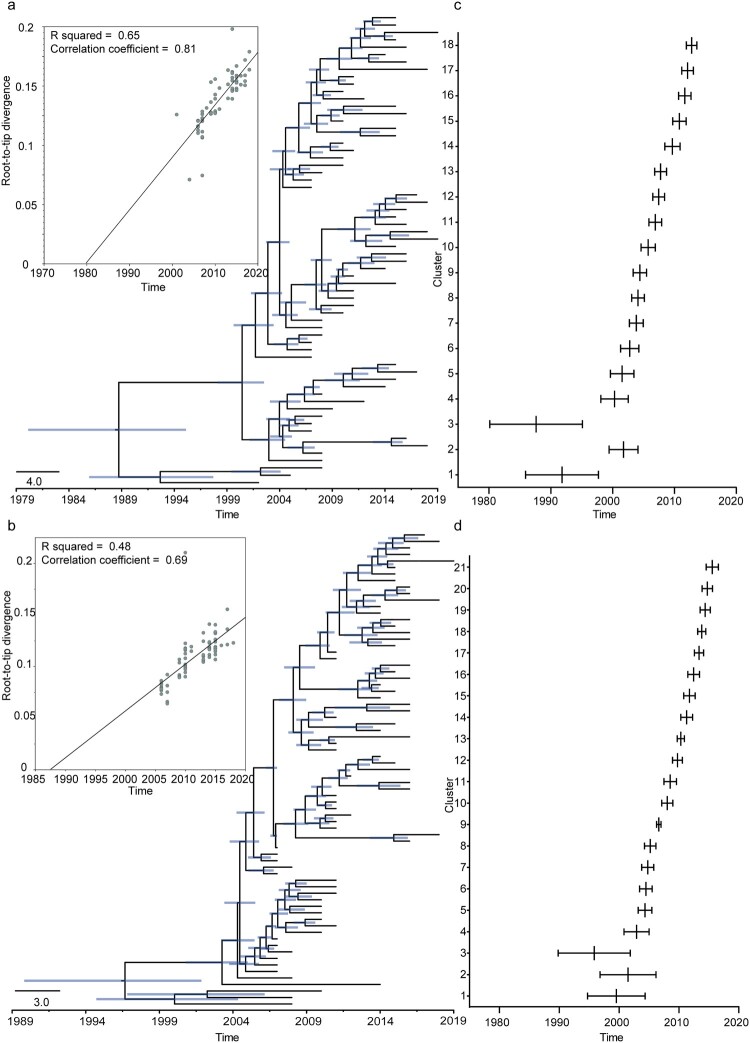
Dynamic time evolution. Phylogenetic tree depicting the inferred ancestry of full-length sequences of (a) H6N6 HA and (b) H6N6 NA genes derived from viruses isolated in China. The line indicates the estimated median age in coalescent analysis, while the horizontal bar represents 95% HPD for the most recent common ancestors. The MCC tree employed FigTree (version 1.5) to display the evolution time. Base compositional data were plotted using GraphPad Prism statistical software, version 3.4.0. The lower and upper values for the complete (c) H6N6 HA and (d) H6N6 NA datasets illustrate the evolution time of each branch. The thick solid line represents the 95% HPD interval of the horizontal line for each branch.

To explore the dynamic evolution of H6N6 viruses in China, we clustered H6 HA gene segments from 462 H6N6 strains isolated from China using a similarity threshold of 98%, resulting in 64 representative viruses. Additionally, we performed clustering on the NA genes from 462 H6N6 strains with a threshold of 99% to identify 73 representative viruses. We conducted an initial analysis of temporal structure using root-to-tip regression, which provided evidence of some temporal (clock-like) structures within the HA (n = 64; data range = 17 years, correlation coefficient = 0.81; R^2^ = 0.65) and NA (n = 73; data range = 12 years, correlation coefficient = 0.69; R^2^ = 0.48) segments of H6N6 viruses (Figures 4a,b). The Bayesian method estimated the rate of nucleotide substitution in H6N6 viruses (using the same datasets as described above) to be 1.68 × 10^–4^ nucleotide substitutions/site/year (95% highest probability density [HPD]) for the HA gene and 1.24 × 10^–4^ substitutions/site/year for the NA gene (Figures 4a,b). According to the data derived from the MCC tree, the minimum value of the second branch was always within the range of the previous branch (Figures 4c,d and Figures S4–S5). These data suggest that the evolution of H6N6 subtype viruses in China is constant and not disrupted by selection pressure.

### Evolutionary pattern of H6N6 viruses in China

**Figure 5.**
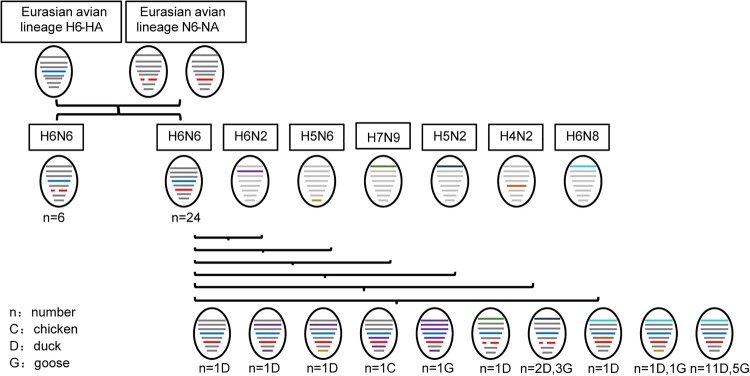
Evolutionary pattern of the isolated H6N6 viruses. Novel H6N6 viruses isolated from domestic chickens, ducks, and geese. The eight gene segments of the viruses are represented by horizontal bars: PB2, PB1, PA, HA, NP, NA, M, and NS.

The evolutionary pattern of H6N6 viruses in China was studied to evaluate the potential threat posed by this dynamically evolving virus. N6 viruses were divided into two lineages based on the deletion of residues 58–68 from the N6 NA gene [34]. Nucleotide identity and phylogenetic tree revealed that eight genes of the 30 H6N6 viruses had been reassorted with six subtypes of viruses: H4N2, H5N2, H5N6, H6N2, H6N8, and H7N9 (Figure 5 and Figure S6). Specifically, for G77 viruses, the PB1 and NS segments exhibited 98.63% and 99.76% identity, respectively, with H6N2 viruses. The PB1 and NS segments of G79 exhibited 98.63% and 99.64% identity with the H6N2 and H5N6 viruses, respectively. The NP and M segments of G70 exhibited 99.46% and 99.80% identity with H6N2 viruses, respectively. The PB2 segments of G73, G81, and G89 viruses exhibited 93.49%, 99.69-99.78%, and 97.58-98.90% identity with H7N9, H5N2, and H6N8 viruses, respectively. For G72 viruses, the PB2 and NP segments exhibited 98.80% and 98.06% identity with H6N8 and H4N2 viruses, respectively. The PB2 and NS segments of G87 viruses showed 98.27-98.29% and 99.52% similarity with H6N8 and H5N6 viruses, respectively. For G65 viruses, PB2, PB1, PA, NP and NS segments exhibited 97.58%, 97.93%, 97.26%, 97.66%, and 98.09% similarity, respectively, with H6N2 viruses. The G97 included eight segments derived from H6N6 viruses (Table 2 and Supplementary Data 3).

**Table 2.** Highest nucleotide identity of the 30 H6N6 AIVs within the database.

Representative strain	Genotype	Segment	The highest nucleotide identity strain	Identity (%)
A/duck/Hunan/HN123/2014	G77	PB1	A/duck/Hunan/S1284/2009(H6N2)	98.63
		NS	A/duck/Guangxi/132/2013(H6N2)	99.76
A/duck/Hunan/HN127/2014	G79	PB1	A/duck/Hunan/S1284/2009(H6N2)	98.63
		NS	A/chicken/Ganzhou/GZ21/2015(H5N6)	99.64
A/chicken/Anhui/AH183/2015	G70	NP	A/duck/Zhejiang/77174/2014(H6N2)	99.46
		M	A/chicken/Zhejiang/77025/2014(H6N2)	99.8
A/goose/Anhui/52/2016	G73	PB2	A/chicken/Ganzhou/GZ79/2016(H7N9)	93.49
A/goose/Anhui/39/2016	G81	PB2	A/chicken/Zhejiang/217/2016(H5N2)	99.69-99.78
A/duck/Hunan/HN209/2017	G72	PB2	A/duck/China/GZ2162/2014(H6N8)	98.8
		NP	A/duck/Guangdong/S4040/2011(H4N2)	98.06
A/duck/Hunan/HN233/2017	G87	PB2	A/duck/China/GZ2162/2014(H6N8)	98.27-98.29
		NS	A/chicken/Ganzhou/GZ21/2015(H5N6)	99.52
A/goose/Hunan/HN268/2017	G65	PB2	A/duck/China/Q032/2013(H6N2)	97.58
** **		PB1	A/Partridge/China/Y015/2013(H6N2)	97.93
** **		PA	A/goose/China/GX1001/2013(H6N2)	97.26
** **		NP	A/goose/Guangxi/128/2013(H6N2)	97.66
** **		NS	A/duck/China/Q032/2013(H6N2)	98.09
A/duck/Hunan/HN288/2017	G89	PB2	A/duck/China/GZ2162/2014(H6N8)	97.58-98.80
A/duck/Hunan/HN277/2017	G97	-	-	-

### The receptor-binding preference and pathogenicity of the representative viruses

To determine whether H6N6 viruses pose a potential pandemic threat, the pathogenicity of the H6N6 virus was evaluated in chickens and mice. The receptor-binding assay of the 10 representative H6N6 viruses indicated that all viruses preferentially bind to SA α-2, 3 receptors (Table 3). The representative strains showed an IVPI value below 1.2, indicating low pathogenicity in chickens (Table 3). Furthermore, the amino acid sequence observed at the cleavage site of these viruses was PQIETR*GLF, confirming their low pathogenicity in the avian population.

**Table 3.** The pathogenicity and receptor-binding preference of the representative viruses.

Viruses	IVPI	HA cleavage site (H3-numbering)	Receptor-binding preference SA α-2, 3 SA α-2, 6	
A/duck/Hunan/HN123/2014	0.00	PQIETR*GLF	Yes	No
A/duck/Hunan/HN127/2014	0.00	PQIETR*GLF	Yes	No
A/chicken/Anhui/AH183/2015	0.00	PQIETR*GLF	Yes	No
A/goose/Anhui/52/2016	0.03	PQIETR*GLF	Yes	No
A/goose/Anhui/39/2016	0.00	PQIETR*GLF	Yes	No
A/duck/Hunan/HN209/2017	0.00	PQIETR*GLF	Yes	No
A/duck/Hunan/HN233/2017	0.00	PQIETR*GLF	Yes	No
A/goose/Hunan/HN268/2017	0.00	PQIETR*GLF	Yes	No
A/duck/Hunan/HN288/2017	0.00	PQIETR*GLF	Yes	No
A/duck/Hunan/HN277/2017	0.00	PQIETR*GLF	Yes	No

**Figure 6.**
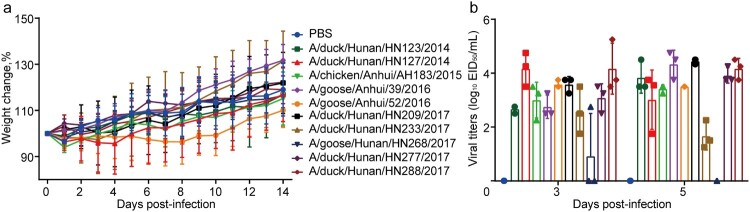
Replication and virulence of representative H6N6 viruses in mice. Eleven mice per group were inoculated intranasally with PBS (control) or 10^6^ EID_50_/ml of the representative H6N6 viruses. (a) Bodyweight changes of the mice were monitored. (b) Viral titers in the lungs of mice. Data represent the mean titers from three mice.

To investigate the potential threat of H6N6 viruses to mammals, we tested the replication and virulence of the representative viruses in mice. After inoculation with the representative viruses, BALB/c mice showed no weight loss or mortality (Figure 6a). As shown in Figure 6b, all 10 viruses replicated well in the lung tissues of mice.

These results indicated that H6N6 viruses could infect mice without prior adaptation.

## Discussion

Worldwide control and surveillance efforts on AIVs have long focused on the H5, H7, and H9 subtypes, owing to their high mortality rates and significant economic losses. However, increasing evidence demonstrates that low pathogenic AIVs deserve more attention because they can potentially infect domestic poultry and mammals by producing pathogenic phenotypes after reassortment. Previous studies suggested that H6N6 viruses contributed NA and internal genes to H5N6 viruses, which have caused 90 human cases since 2013 [10,35]. The H6N6 virus can infect mammals, such as mice and swine [18,20,36]. However, no information is available regarding the evolutionary patterns of H6N6 viruses. In the present study, we conducted phylogenetic analysis and ML evolutionary analysis of the H6N6 viruses in China, established a molecular clock tree for the HA and NA genes of H6N6 viruses, and detected the evolutionary pattern and pathogenicity of H6N6 viruses.

Previous studies have demonstrated that the length of the NA stalk affects the biological characteristics of the virus [37,38]. The deletion in the NA stalk disrupts the balance between HA and NA, reduces the function of NA, and inhibits the replication of influenza viruses [39]. Our results revealed that the NA protein from 6/7 Anhui strains possessed an 11-amino acid deletion within the NA protein, which was absent in the Hunan strains. Additional investigation are required to elucidate the influence of NA deletion on the biological characteristics of H6N6 viruses.

It has been revealed that novel H6N6 replaced H6N2 viruses and has become the most frequently detected subtype among H6 subtype viruses since 2009 in Eastern China [17]. In this study, the HN268 (H6N6) strain occurred between 2006 and 2007 with PB2, PB1, PA, NP, and NS gene segments reassorted with H6N2 viruses. The PB1 and NS segments of HN123 (H6N6) from 2007 to 2009 were reassorted from H6N2 viruses (Figure 5a,b). These results indicate that, as early as 2006, H6N6 viruses had frequently reassorted with H6N2 viruses and eventually replaced H6N2 to become the most prevalent strain of the H6 subtype in China.

Based on the bootstrap values of the ML trees, 98 genotypes were identified (Figure 3 and Figure S2). Among the 98 genotypes, only six (G20, G24, G25, G49, G69, and G90) appeared in the adjacent two years, while the remaining genotypes were distributed within only one year. The results of the genotype distribution based on isolation time indicated that the genotypes were only isolated in one or two years and quickly disappeared. These results indicated that H6N6 viruses were undergoing continuous gene replacement and evolution in China.

**Figure 7.**
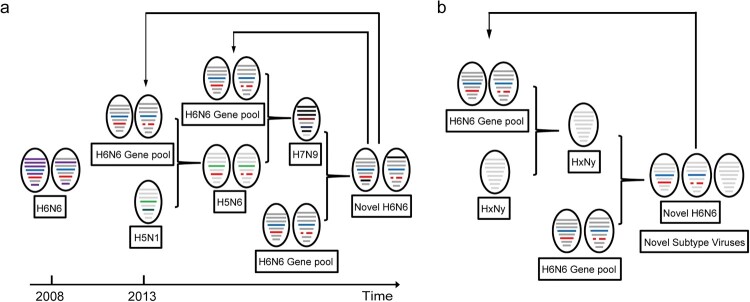
The evolutionary pattern of H6N6 viruses circulating in China. The eight gene segments of the viruses are represented by horizontal bars: PB2, PB1, PA, HA, NP, NA, M, and NS. (a) The evolutionary model of H6N6 viruses with H5N6 and H7N9 viruses was predicted by combining the results of the previous study and the present study in our laboratory. (b) A hypothetical evolutionary pattern of H6N6 viruses circulating in China.

Previous study has reported that H6N6 viruses contribute NA and internal genes to H5N6 viruses [9]. H7N9 viruses have changed several gene segments through reassortment with H6N6 viruses [11,12]. Moreover, the present study revealed that both H5N6 and H7N9 viruses have contributed to the emergence of novel H6N6 viruses (Figure 5). Collectively, the previous research and the present data suggested that the H6N6 viruses formed an evolutionary circle with the H5N6 and H7N9 viruses through reassortment (Figure 7a). More importantly, H6N6 viruses may acts as an intermediary in the evolution of H5N6, H6N6, and H7N9 viruses (Figures 7b).

Previous studies have shown that the H6 subtype viruses belonging to the ST2853-like clade can infect swine, mice, and human lungs without prior adaptation [14,18–21]. In the present study, 30 isolated strains belonged to the ST2853-like clade. Consistently, the 10 strains of H6N6 viruses can infect mice without prior adaptation, suggesting that the avian-originated H6N6 viruses have the potential to cross the species barrier to infect mammals.

Migratory wild birds play a crucial role in the global distribution of influenza viruses and waterfowls are the significant natural reservoir for influenza viruses. Compared to wild birds and waterfowls, chickens have more opportunities to come in contact with humans. While the role of chickens in the evolution of IAV is mainly local, they play a crucial role as a source of human infection with AIVs, and may act as intermediate hosts in the transmission of influenza viruses from avian to human populations [40,41]. Our data revealed that ducks were the most important reservoir (75.51%) for H6N6 isolates, while chickens accounted for 15.31% of the H6N6 isolates. The chicken-derived H6N6 virus (AH183) can infect mice without prior adaptation. Therefore, the role of chickens in the evolution of low pathogenic IAV deserves more concern.

The present study provides a comprehensive overview of the evolutionary dynamics of H6N6 viruses. Our study revealed the ongoing circulation of H6N6 viruses in domestic farms and LPMs, which could potentially become circulating viruses and participate in the evolution of influenza viruses. The results revealed that the H6N6 viruses showed low pathogenicity in chickens and mice. However, avian H6N6 viruses replicate well in mice lungs without prior adaptation. Therefore, it is necessary to emphasize the importance of monitoring a wide range of H6 avian influenza subtypes in different hosts and regions.

## Supplementary Material

Supplement_Data

Description_of_Additional_Supplementary_Files
