# Supplementary material for: Evolution of H6N6 viruses in China between 2014 and 2019 involves multiple reassortment events
Source: Emerg Microbes Infect. 2024 Apr 6;13(1):2341142. doi: 10.1080/22221751.2024.2341142 (PMC11041520; doi:10.1080/22221751.2024.2341142)
Supplement: Description_of_Additional_Supplementary_Files [file TEMI_A_2341142_SM6972.pdf]

Description of Supplementary Files

Evolution of H6N6 viruses in China between 2014 and 2019 involves multiple reassortment events

Yingying Du[a#], Jun Xia[b#], Zhengxiang Wang[a], Jie Xu[a], Yanhong Ji[a], Yinghong Jin[b], Ling Pu[c], Shuai Xu*[a].

[a] State Key Laboratory for Animal Disease Control and Prevention, College of Veterinary Medicine, Lanzhou University, Lanzhou Veterinary Research Institute, Chinese Academy of Agricultural Sciences, Lanzhou 730000, China;

[b] Institute of Veterinary Medicine, Xinjiang Academy of Animal Sciences, Urumqi 830013, China;

[c] Guizhou Institute of Animal Husbandry and Veterinary Science, Guizhou 550005, China.

Corresponding author:

Shuai Xu, E-mail: xushuai@caas.cn

Supplementary Materials and methods

Receptor binding specificity

The receptor-binding specificity of virus was performed according to the manual from the WHO using 1% chicken red blood cells (cRBCs, expressing both α2,3-SA and α2,6-SA receptors), 1% resialylated cRBCs (α2,6-SA receptors), and 1% sheep red blood cells (sRBCs, α2,3-SA receptors) [1]. The resialylated cRBCs were generated by treating cRBCs with 2, 3-sialidase (Takara Bio, China) to remove the α2,3-SA receptors from the cRBCs. Specifically, we added 10 μl 2,3-sialidase (with 50 mu/μL concentration) to 90 μL 10% cRBCs suspension, mixed gently, and incubated them for 15 min at 37°C. Then, the treated cRBCs were washed twice by phosphate buffer saline (PBS) and centrifuged at 400 × g for 5 min each time. The sediments were used to prepare the 1% resialylated cRBCs by PBS.

References

[1] WHO. Manual for the laboratory diagnosis and virological surveillance of influenza 2011. Available from: https://www.who.int/publications/i/item/manual-for-the-laboratory-diagnosis-and-virological-surveillance-of-influenza

Supplementary Figure Legend

Supplementary Figure 1. Alignment of the 50-70 amino acid of NA stalk region of 30 H6N6 viruses.

Supplementary Figure 2. The ML trees of HA, NA, PB2, PB1, PA, NP, M, and NS genes of the 198 H6N6 viruses. The red taxon branch represents the strains deposited in this study.

Supplementary Figure 3. ML tree of H6N6 HA gene. The strains isolated in this study are highlighted in red on the phylogenetic tree. The genotype distribution of the complete genomes of H6N6 viruses was based on a group of bootstrap values. Each vertical bar represents a gene group.

Supplementary Figure 4. Phylogenetic tree depicting the inferred ancestry of full-length sequences of H6N6 HA genes derived from viruses isolated in China. The line indicates the estimated median age in coalescent analysis, while the horizontal bar represents 95% HPD for the most recent common ancestors. The MCC tree employed FigTree (version 1.5) to display the evolution time. Base compositional data were plotted using GraphPad Prism statistical software, version 3.4.0.

Supplementary Figure 5. Phylogenetic tree depicting the inferred ancestry of full-length sequences of H6N6 NA genes derived from viruses isolated in China. The line indicates the estimated median age in coalescent analysis, while the horizontal bar represents 95% HPD for the most recent common ancestors. The MCC tree employed FigTree (version 1.5) to display the evolution time. Base compositional data were plotted using GraphPad Prism statistical software, version 3.4.0.

Supplementary Figure 6. Phylogenetic tree of the reassorted viruses. The scale bar represents the number of nucleotide substitutions per site. The H6N6 viruses reported in the present study are marked in red.
